# Bifunctional MXene quantum dots-coated bimetallic Prussian blue analogues for sensitive sensing and accurate localization imaging of miRNAs in living cells

**DOI:** 10.1016/j.mtbio.2025.101747

**Published:** 2025-04-15

**Authors:** Qiannan You, Panyong Wang, Tongtong Zhu, Zixuan Jia, Zhimin Chang, Li Li, Wen-Fei Dong

**Affiliations:** a Department of Biomaterials and Stem Cells, Suzhou Institute of Biomedical Engineering and Technology, Chinese Academy of Science, Suzhou, 215163, PR China; b School of Biomedical Engineering (Suzhou), Division of Life Sciences and Medicine, University of Science and Technology of China, Hefei, 230026, PR China

**Keywords:** MicroRNAs sensing, Living cells imaging, Bimetallic prussian blue analogue, MXene quantum dot

## Abstract

MicroRNAs (miRNAs) are involved in multiple cellular processes and play a critical role in clinical diagnosis. In-situ spatiotemporal imaging of miRNAs in living cells is tightly linked to the carcinogenesis and development of malignant tumors. Herein, we proposed a bifunctional nanosystem-based MXene quantum dots-coated bimetallic Prussian blue analogues (Co-Mn PBA@MQDs) to execute in-vitro sensing and intracellular imaging of miRNA in living cells. The 3D nanostructures of Co-Mn PBAs were regulated to slow down the coordination reaction rate by controlling the diffusion of metal clusters and ligand precursors, thereby anchoring MQDs as the carriers of DNA probes. The resulting Co-Mn PBA@MQDs nanoparticles with miRNA recognition ability exhibit excellent electrocatalytic and photoluminescence properties for target miRNA analysis. It reached miRNA detection limit of 0.37 fM (S/N = 3) with a wide linear range of 1 fM to 1 nM, and allowed distinguish family members without additional complex modifications. Meanwhile, DNA probe adsorbed on Co-Mn PBA@MQDs can provide delivery capacity for intracellular miRNA location, resulting in the in-situ monitoring and imaging of miRNA with deregulated expression levels in cancer cells. With these advantages, the developed strategy provides a paradigm for the rational design of the miRNA analysis system, which is expected to be widely applied to disease diagnosis and further theragnostic fields.

## Highlights

• MQDs were integrated into bimetallic PBA frameworks via coordination restriction.
• The nanosystem was used to detect miRNA without any amplification steps.
• The nanosystem exhibited efficient location ability toward intracellular miRNAs.

## Graphical abstract

**Figure:**
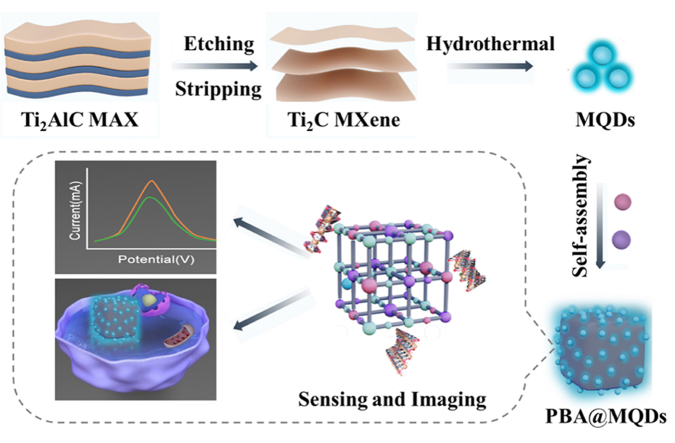

## 1 Introduction

MicroRNAs (miRNAs) are endogenous noncoding RNA with a length of about 22 nucleotides, which participate in the regulation of various cellular processes including cell differentiation, proliferation, and apoptosis of organisms, etc [[1], [2], [3]]. Dysregulated expression of miRNAs is closely associated with the occurrence and development of various diseases, such as the abnormal regulation of miRNAs express different patterns with the occurrence and development of tumors, involving the mutation and abnormal expression of multiple genes or specific miRNAs [[4], [5], [6]]. Therefore, miRNAs are recognized as valuable biomarkers for early clinical diagnosis and monitoring of cancers, where the accurate and efficient detection of miRNAs is essential [[7], [8], [9]]. In particular, the direct visualization of miRNA cellular location is of great significance to understanding pathological and physiological conditions [[10], [11], [12]]. However, several challenges limit miRNA analysis, including the heterogeneous origins of miRNAs, low abundance, and similar sequences. As a result, developing an accurate, sensitive, and specific method for sensing and intracellular imaging of miRNAs is critically needed.

At present, a variety of analytical techniques have been developed for the accurate and sensitive assay of miRNAs, e.g. nano-flash enzyme amplification based on molecular beacons, enzyme-free DNA loop, catalytic hairpin assembly hybrid chain reaction, and entropy-driven reaction [[13], [14], [15], [16]]. Although these technologies achieved successful detection and imaging of miRNAs, most of them suffer from cumbersome probe design, high-cost equipment, and rigid operational procedures. Electrochemical biosensors have been developed for clinical diagnosis owing to their miniaturization of electrodes, rapid signal response, and mature modification nanotechnology [[17], [18], [19]]. The effective combination of molecular probes and complex background interference are essential points to be considered when fabricating the biosensing interface of electrochemical biosensors, which are still hampered by multiple detection steps and are complicated. Therefore, it is significant to develop a simple, rapid, and efficient nanosystem with not only excellent electrocatalytic performance but also efficiently visualize the target miRNA in the complex intracellular environment.

Metal-organic frameworks (MOFs) with adjustable pore structure, large porosity, and rich functionality have witnessed wide applications in biosensors, drug delivery, and photoelectrocatalysis [[20], [21], [22]]. Among the developed crystalline MOF systems, Prussian blue analogues (PBAs) exhibit great potential in electrocatalysis with excellent stability, 3D open frameworks, and controllable nanostructures [[23], [24], [25]]. These features would make it easier for the load of molecular and diffusion of charge carriers, thus ensuring high-sensitive response performance. In particular, the bimetallic PBA possesses different redox-active sites and equilibrium charge vacancies, which increase catalytic activity and cycle properties to construct biosensors. Given that the formation of PBA with a homogeneous crystalline structure is related to the uncontrollable nucleation and reaction rate during the coordination process, the development of a synthetic strategy by endowing a controllable diffusion rate is critical for delicate structure construction.

MXene is commonly obtained via etching A element layers from MAX precursor phases with a general formula, M_n+1_X_n_T_x_ (where M is early transition metal, n = 1∼3, X represents C and/or N, T_x_ is surface termination group, and A is A-group element) [[26], [27], [28]]. MXene shows good electrical conductivity, adsorption capacity for biorecognition probes, and hydrophilic properties, which expand their extensive applications, especially in sensing detection [[29], [30], [31]]. However, the transverse dimension and low photoluminescence of MXene limits its direct application in biological imaging [32]. Recently, theoretical and experimental studies indicated that a synthetic pathway reducing 2D MXene to 0D MXene quantum dots (MQDs) is feasible for the endowment of tunable photoluminescence [[33], [34], [35]]. Based on the distinctive confinement effect of 0D structure, MQDs have emerged as promising optical materials with the intrinsic characteristics of aqueous colloidal stability, ultrasmall size, and photoluminescence effect, which would be promising candidate materials for the intracellular imaging of miRNAs.

**Scheme 1:**
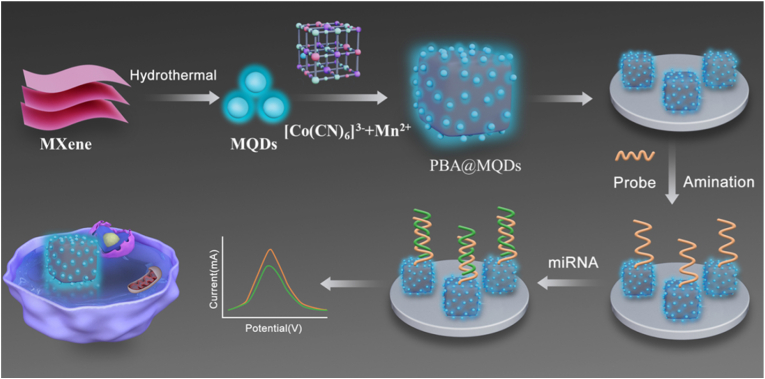
Schematic illustration of the Co-Mn PBA@MQDs nanoparticles for the electrochemical detection and visualization location of miRNA in living cells.

Herein, we design a novel nanosystem based on Co-Mn PBA@MQDs nanoparticles with DNA probe assistance for electrocatalysis sensing and cellular miRNA imaging (Scheme 1). MQDs with unique photoluminescence as nanocarrier were obtained via a facile hydrothermal process, following confined-coordination decreased bimetallic Co-Mn PBA growth rate by regulating the metal cluster and ligand diffusion reaction. Under the electrostatic effect, MQDs and Co-Mn PBAs combined to form a core-shell structure. Importantly, the adsorption of MQDs not only inhibited the self-aggregation of PBAs, and did not influence the nucleation of PBAs to grow into uniform nanocubes. The as-prepared Co-Mn PBA@MQDs nanoparticles exhibit excellent electrocatalysis, photoluminescence, and available active sites for both electrical-optical signal generation and capture DNA probe immobilization. With the DNA probe hybridization, the functionalized Co-Mn PBA@MQDs nanoparticles as electrode modification interface could specifically anchor target miRNA to induce current response, which indicated the satisfactory preparation of a sensitive miRNA-141 biosensor. Meanwhile, the as-prepared nanoparticles were employed as molecular imaging agents, inducing the localization and visualization of miRNA in living cells. Accordingly, the Co-Mn PBA@MQDs-based nanosystem is expected to be a promising tool for early cancer diagnosis and prognosis monitoring.

## 2 Experiments

### 2.1 Synthesis of Ti_2_CT_x_ MXene quantum dots (MQDs)

Multi-layered Ti_2_CT_x_ MXene was first synthesized by a wet etching method according to previous works. Typically, 0.666 g LiF was dispersed in 10 mL HCl (6 M) and stirred for 30 min. Then, 1 g Ti_2_AlC MAX precursor was added and reacted for 36 h at 35 °C. The solution was washed several times by centrifugation and dried overnight in a vacuum oven at 60 °C.

A hydrothermal process was carried out for the fabrication of Ti_2_CT_x_ MXene quantum dots (MQDs). 200 mg of multi-layered Ti_2_CT_x_ MXene was dissolved in 20 mL DI water and ultrasonic for 30 min. The pH of the mixture was adjusted to 9.0 with NH_3_·H_2_O. Then, the suspension was transferred into a 50 mL Teflon-lined autoclave and heated at 120 °C for 6 h. The MQDs could be obtained by 0.22 μm membrane filtration.

### 2.2 Synthesis of Co-Mn PBA@MQDs

In a typical synthesis, 0.01 M of MnSO_4_ and 0.1 M of KCl were dissolved in 50 mL DI water to form solution A. 0.01 M of K_3_ [Co(CN)_6_] and 0.1 M of KCl were dissolved in 50 mL DI water to form solution B. Then, solutions A and B were injected into solution C (MQDs) with an injection rate of 200 μL/min under magnetic stirring at 35 °C. Finally, the resulting suspension was washed with water and ethanol and dried in a vacuum oven at 60 °C for 12 h.

### 2.3 In vitro detection of miRNA

10 mg PEI and 2 mg Co-Mn PBA@MQDs were dissolved in 2 mL DI water at room temperature, and the mixture was stirred for 4 h to obtain the aminated composite particles (NH_2_-PBA@MQDs). Then, 10 μL NH_2_-PBA@MQDs was doped on the Au disk electrode surface (Φ = 3 mm) and dried under N_2_ atmosphere. After drying, the as-prepared electrode was activated by a mixture solution containing 0.3 M EDC and 0.6 M NHS at 4 °C for 2 h. Subsequently, 5 μL of 5 μM probe was immobilized on the modified electrode for 12 h at 4 °C to construct the probe/NH_2_-PBA@MQDs, followed by W-buffer rinsed to remove the unfixed residual probe. Finally, the unreacted activation sites of the probe/NH_2_-PBA@MQDs modified electrode were passivated with 5 μL of 10 mM MCH solution for 1 h at 4 °C. For miRNA sensing, 10 μL miRNA-141 solution with different concentrations was prepared with H-buffer, then the probe/NH_2_-PBA@MQDs modified electrodes were immersed in the solution and incubated at 37 °C for 6 h to obtain miRNA/probe/NH_2_-PBA@MQDs modified electrodes.

### 2.4 Cell culture and cytotoxicity assay

A549, Hela, and 3T3 cells were cultured in an incubator containing 5 % CO_2_ at 37 °C, and the culture medium was a RPMI-1640 medium containing 10 % fetal bovine serum (FBS), 100 μg/mL streptomycin, and 100 U/mL penicillin. For imaging the miRNA in living cells, MTT Cell Proliferation-Toxicity Test (MTT assay) was carried out to evaluate the cytotoxicity of the PBA@MQDs. Briefly, these three kinds of cells were plated in a 96-well cell culture plate (10^4^ cells per well) and incubated for 24 h. Afterward, the plate was washed three times with PBS and incubated with different concentrations of PBA@MQDs nanoparticles for another 36 h. After the incubation, the cells were still washed with PBS three times and cultured with MTT solution at 37 °C for 4 h. The cell viability was assessed by reading the absorbance of the microplate reader at 490 nm.

### 2.5 Imaging of intracellular miRNA

A549 Cells were seeded in 35 mm confocal dishes and cultured to a confluency of 70–80 %, then incubated with 100 μL RPMI-1640 medium containing probe/NH_2_-PBA@MQDs nanoparticles for 6 h at 37 °C. By contrast, Hela cells and 3T3 cells were incubated with probe/NH_2_-PBA@MQDs nanoparticles in the same cell culture medium. After being incubated for 6 h, they were washed with PBS three times, and fluorescence images of cells were visualized by a laser scanning confocal microscope.

## 3 Results and discussion

### 3.1 Preparation and characterization of Co-Mn PBA@MQDs nanoparticles

**Fig. 1:**
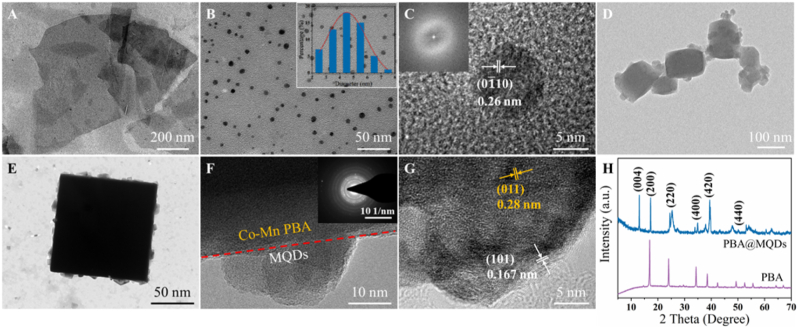
TEM images of (A) Ti_2_CT_x_ MXene and (B) MQDs. Inset: Corresponding size distribution. (C) HRTEM image of MQDs. Inset: Corresponding SAED pattern. (D–F) TEM, and (G) HRTEM images of Co-Mn PBA@MQDs nanoparticles, the inset is the corresponding SAED pattern. (H) XRD patterns of Co-Mn PBA and Co-Mn PBA@MQDs nanoparticles, respectively.

Ti_2_CT_x_ MXene, a member of the sensing element, possesses metallic conductivity and hydrophilicity making them promising candidates for biomedical and electrochemical sensing. Moreover, transforming MXene from 2D nanosheets into 0D quantum dots can confer the luminescence properties of ultrasmall size via a facile hydrothermal process. Briefly, the Ti_2_AlC MAX precursor underwent an acid-etching treatment assisted with organic solvent intercalation to convert it into 2D flakes known as Ti_2_CT_x_ MXene. The scanning electron microscopy (SEM) and transmission electron microscopy (TEM) images clearly shows layered structure of Ti_2_CT_x_ MXene was exfoliated successfully from MAX precursor (Fig. S1), and then layers of MXene nanosheets were obtained with a lateral dimension of ∼600 nm (Fig. 1A). Subsequently, the MXene nanosheets were further transformed into MQDs via a hydrothermal method in an ammonia aqueous solution as follows:

MXene + H_2_O → Titanium Hydroxide

Titanium Hydroxide + 2NH_3_·H_2_O → TiO_2_^−^ + 2NH_4_^+^ + H_2_O

**Fig. 2:**
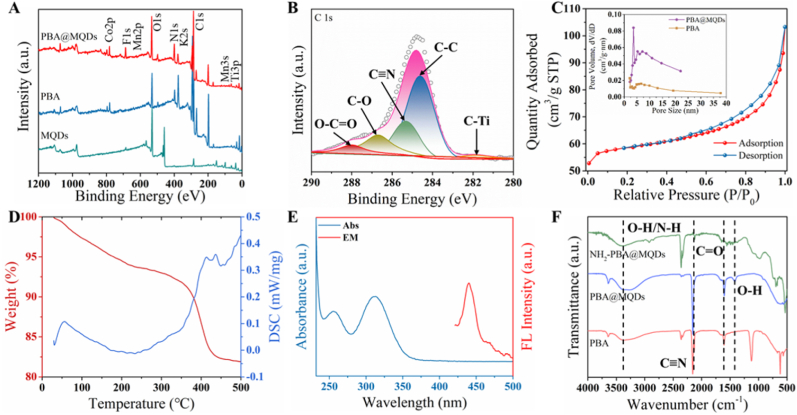
XPS spectra of (A) MQDs, PBA, and PBA@MQDs. (B) C 1s from PBA@MQDs. (C) N_2_ adsorption-desorption isotherms of PBA@MQDs with inserted the corresponding pore size distribution of PBA and PBA@MQDs. (D) TG curve and (E) UV–vis absorbance and emission curves of PBA@MQDs, respectively. (F) FT-IR spectra of PBA, PBA@MQDs, and NH_2_-PBA@MQDs, respectively.

The TEM image (Fig. 2B) revealed that the average size of the MQDs was uniform and the size distribution can be calculated as 4.55 nm. The crystal structure of the as-prepared MQDs was further investigated by high-resolution transmission electron microscopy (HRTEM), in which the lattice stripes with inner plane spacing of 0.26 nm correspond to the (0_110) plane of Ti_2_CT_x_ MXene (Fig. 2C). To further verify forming structure of MQDs, X-ray diffraction (XRD), Fourier transform infrared spectra (FT-IR), and X-ray photoelectron spectroscopy (XPS) were used to characterize its lattice structure and functional groups. As shown in Fig. S3A, the characteristic peak of (002) shifted from 13.1° of Ti_2_AlC MAX to 7.3° of Ti_2_CT_x_ MXene, indicating the expansion of adjacent MXene layers with the interlayer spacing of 1.21 nm. The presence of a diffraction peak at around 24.1° suggests the formation of Ti-O^-^, showing the successful synthesis of MQDs. The FT-IR spectrum (Fig. S3B) of MQDs exhibit chemical bond stretching vibration, including the -OH at 3407 cm^−1^, C=O at 1639 cm^−1^, and -NH at 3100 cm^−1^, indicating the as-prepared MQDs were functionalized with -NH groups during hydrothermal treatment. The functionalization and formation of MQDs were confirmed through XPS. Fig. S3C shows two predominant Ti 2p3/2 peaks at 458.1 eV and 465.2 eV, which belong to the Ti-C bond of the MQDs core and the Ti-O bond embedded in MQDs surface, respectively. Therefore, it is confirmed that MQDs with well-defined crystal structures of pristine MXene can be formed after the hydrothermal process.

**Fig. 3:**
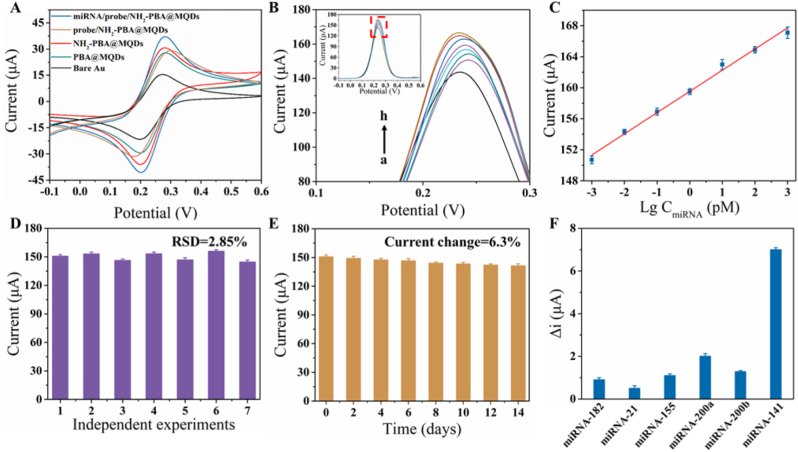
(A) CV behaviors of bare Au disk electrode, PBA@MQDs, NH_2_-PBA@MQDs, probe/NH_2_-PBA@MQDs, and miRNA/probe/NH_2_-PBA@MQDs modified electrodes, respectively. The concentration of miRNA-141 was 1 fM. (B) SWV responses of the miRNA/probe/NH_2_-PBA@MQDs modified electrodes with different concentrations of miRNA-141. The concentration of curves a to g is 0, 1 fM, 10 fM, 100 fM, 1 pM, 10 pM, 100 pM, 1 nM. (C) Linear relationship between peak current and the miRNA-141 concentration. (D) Reproducibility of 7 independent miRNA/probe/NH_2_-PBA@MQDs modified electrodes (n = 3). The concentration of miRNA-141 was 1 fM. (E) Long-term stability in 14 days. The concentration of miRNA-141 was 1 fM. (F) Selective determination of the probe/NH_2_-PBA@MQDs. The concentrations of miRNAs were 1 fM.

Co-Mn PBA@MQDs nanoparticles were prepared via a facile co-precipitation method, where the precursor hexacyanocobaltate (Ⅲ) and manganese ion (Ⅱ) were introduced to MQDs solution, then Co-Mn PBA was coordinated to obtain Co-Mn PBA@MQDs. As shown in Fig. 1D, Co-Mn PBA@MQDs presented regular nano-cubic with a uniform diameter of ∼100 nm, while clear adhesion layer can be observed on the periphery of nanoparticle (Fig. 1E). As determined by HRTEM and SAED, the regular arrangement of diffraction spots can be clearly observed in Fig. 1F, and the lattice spacing of 0.280 nm and 0.167 nm correspond to the (011) plane of PBA and the (101) plane of MQDs, respectively (Fig. 1G). The ζ-Potential (Fig. 3F) further confirmed the formation of PBA@MQDs nanoparticles. The ζ-Potential of MQDs was negatively charged with −19.6 mV due to their abundant hydroxyl groups, while the ζ-Potential value of PBA@MQDs composites increased to −11.7 mV with the combination of Co-Mn PBA and MQDs. It demonstrated the existence of electrostatic interactions between Co-Mn PBA and MQDs, which enables MQDs to be stably anchored on the PBA surface. The nanoparticle evolution during the preparation process was performed by XRD, XPS, and FT-IR. The XRD results indicated that the PBA@MQDs retained their original crystalline structure of PBA and the (004) crystal plane of MQDs (Fig. 1H). The elemental mapping images further explicated the successful preparation of the Co-Mn PBA@MQDs (Fig. S4).

We further investigate the respective elemental compositions and chemical environment of MQDs, PBA, and PBA@MQDs by XPS (Fig. 2A). Prior to the modification of MQDs, the Co and Mn peaks can be observed following the coordinate of PBA nanoparticles. Additionally, the C 1s spectrum in Fig. 2B exhibited peaks at 281.8 eV, 284.6 eV, 285.2 eV, 286.7 eV, and 288.1 eV corresponding to C-Ti, C-C, C ≡ N, C-O, and O-C=O, suggesting the Co-Mn PBA nanoparticles were successfully absorbed with MQDs following coordination self-assembly. Furthermore, as depicted in Fig. S5, the Co 2p spectrum can be deconvoluted into two pairs of peaks attributed to Co 2p_1/2_ (796.3 eV) and Co 2p_3/2_ (781.5 eV). The Mn 2p showed two peaks at 641.4 eV and 653 eV, representing the characteristic Mn 2p_3/2_ and Mn 2p_1/2_, respectively. Fig. 2C demonstrated the type-Ⅳ N_2_ adsorption-desorption isotherms with a hysteresis loop in the relative pressure range of 0.7–1.0 to indicate that the PBA@MQDs nanoparticles possess mesoporous structure. The Brunauer-Emmett-Teller (BET) surface area of PBA@MQDs can be calculated to be 183.7 m^2^ g^−1^, which is beneficial to the rapid transparency of electrons and electrolytes to accelerate the overall electrochemical process. To evaluate the chemical stability of PBA@MQDs nanoparticles, the thermogravimetric analysis (TGA) was carried out under an N_2_ atmosphere with a heating rate of 5 °C min^−1^. As shown in Fig. 2D, a slight weight loss appeared at ca. 210 °C, which is ascribed to the loss of crystalliferous water. Then, the decomposition of PBA@MQDs initiates around 370 °C and turns stable at ∼440 °C. In contrast, an obvious weight loss of 92.6 % was shown at about 250 °C of MQDs in Fig. S5D, indicating the enhancement of thermal stability by PBA-doping.

The optical properties of the as-prepared PBA@MQDs were performed by Ultraviolet absorption spectroscopy (UV–vis) and fluorescence spectrophotometry in Fig. 2E. We observed the absorption typical peaks at 260 nm and 320 nm from MQDs and Co-Mn PBA, respectively. And photoluminescence (PL) spectrum of PBA@MQDs with strong emission at 440 nm was obtained under 350 nm excitation, indicating the possibility of bio-imaging applications. Before the as-prepared PBA@MQDs were used for miRNA sensing, amino functional modification was carried out for target aptamer anchoring. As shown in Fig. 2F, the identical stretching vibrations of C=O at 1602 cm^−1^, C ≡ N vibration of Co^3+^-CN-Mn^2+^ at 2160 cm^−1^, -OH among water molecules and the N-H vibration at 3410 cm^−1^, suggesting the successful synthesis of PBA@MQDs as well as surface amination.

### 3.2 Sensing miRNAs in vitro

Cyclic voltammetry (CV) and electrochemical impedance spectroscopy (EIS) were applied to investigate the electrochemical properties of the constructed electrodes. As shown in Fig. S6A, PBA@MQDs modified electrode showed a pair of redox peaks at 0.28 V and 0.20 V with the superior peak intensity. This indicated that the PBA@MQDs composite nanoparticles possessed lower barrier electronic transfer and high electrocatalytic performance, which is consistent with EIS results in Fig. S6B. Besides, we can calculate that the effective specific area of the PBA@MQDs modified electrode has increased by 2.7 times vs. bare Au electrode, enabling immobilize more biological molecular probes (Fig. S6F). The CV tests were further used to electrochemically estimate the electrode surface behaviors during the modified process and analyze the feasibility of miRNA-141 sensing. As shown in Fig. 3A, the aminated modified PBA@MQDs (NH_2_-PBA@MQDs) showed increased redox currents with constant potential positions, which suggested that the decoration of NH_2_ does not affect the electrochemical behavior of PBA@MQDs, but can improve electron migration of the modified electrode. When the probe was immobilized, the current response of probe/NH_2_-PBA@MQDs was decreased due to the presence of non-conductive e oligonucleotides. Similarly, the feasibility of sensing miRNA-141 with probe/NH_2_-PBA@MQDs modified electrode was investigated from the change of redox peak. As expected, a visible electrochemical response signal was observed after miRNA-141 was hybridized with probe, showing the potential for target RNA sensing.

To examine the optimal performance of the PBA@MQDs modified electrodes for miRNA-141 sensing, a series of parameters were analyzed by square wave voltammetry (SWV). First, the sensitivity of the probe/NH_2_-PBA@MQDs modified electrode for the detection of target miRNA depends primarily on the immobilization efficiency of probe. Fig. S7A showed that the SWV peak current reached maximum response at 5 μM. Then, we studied the incubation time of NH_2_-PBA@MQDs modified electrode with probe and miRNA-141 with probe/NH_2_-PBA@MQDs modified electrode and found that the optimum detection signal can be achieved at 12 h and 6 h, respectively. In addition, the pH value of H-buffer solution used for miRNA-141 hybridization was also investigated, and the detection signal was optimal as the H-buffer pH up to 7.4.

Given the optimal experimental parameters, the analytic capability for miRNA-141 of the as-prepared electrode was further assessed in vitro. As shown in Fig. 3B, a gradual increase in SWV current responses as the miRNA-141 concentrations increased. The logarithmic relationship (Fig. 3C) between peak current and miRNA-141 concentration over the range from 1 fM to 1 nM was built and the correlation equation is I = 2.73 + 159.5 lgC_miRNA_ (R^2^ = 0.994) and the limit of detection (LOD) was estimated to be 0.37 fM (S/N = 3). Afterward, the performance of the probe/NH_2_-PBA@MQDs modified electrode for detecting miRNA-141 was evaluated under the same optimal experimental conditions. As shown in Fig. 3D, the SWV peak currents of 7 independent probe/NH_2_-PBA@MQDs modified electrodes with different batches were performed to characterize the reproducibility of the as-prepared electrodes. The RSD can be calculated as 2.85 %, implying the excellent reproducibility of the electrodes. Aside from excellent reproducibility, electrochemical stability is another important capability for miRNA sensing. Thus, we conducted an enduring stability test by storing the miRNA/probe/NH_2_-PBA@MQDs modified electrode for 14 days (Fig. 3E). By contrast, the current responses still maintained 93.7 % of the initial current on the 14th day, showing good long-term stability of the prepared electrode. Furthermore, a series of miRNAs including miRNA-182, miRNA-21, miRNA-155, miRNA-200a, and miRNA-200b were selected to investigate the selectivity of probe/NH_2_-PBA@MQDs modified electrodes. As illustrated in Fig. 3F, compared to the recorded signals of miRNA-141 there were 71.4 % and 81.7 % declines in the response signals for mismatched miRNA-200a and miRNA-200b, respectively, while other miRNAs led to nearly negligible current change. Therefore, the interference of other miRNAs on the detection signal can be ignored, which indicates that the electrochemical sensor has good mismatch discrimination ability. As a comparison, this as-prepared biosensor achieved detection liner range and LOD is better than that of most reported works (Table S3).

In order to evaluate the practical performance of our method, we used different concentrations of spiked miRNA-141 in real serum samples to verify sample recovery efficiency of the as-prepared biosensor. As presented in Table S4, a sound recovery of 95.6 %–101.2 % demonstrated that the proposed biosensor had potential applications in real clinical samples.

### 3.3 Intracellular imaging of miRNAs

Benefiting from the excellent performance of probe/NH_2_-PBA@MQDs in vitro, the function of these composite nanoparticles in living cells for target miRNA imaging was further analyzed. Considering the essential biocompatibility for cell imaging, the cytotoxicity assay was performed to study the feasibility of the probe/NH_2_-PBA@MQDs nanoparticles for imaging intracellular miRNA-141. From the MTT Cell Proliferation-Toxicity Test (MTT assay) in Fig. S9, the viability of A549 cells, Hela cells, and 3T3 cells all remained above 80 % after incubated for 36 h with different concentrations of nanoparticles, suggesting that the nanoparticles have potential application in cellular cultures with good biocompatibility. Thus, we applied the probe/NH_2_-PBA@MQDs nanoparticles to incubate with A549 cells for imaging miRNA-141.

**Fig. 4:**
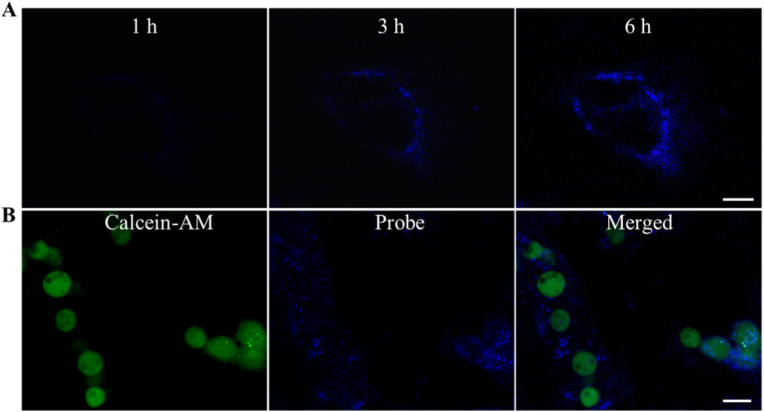
The average fluorescence intensity of laser scanning confocal microscopy images as shown in (A) A549 cells treated with probe/NH_2_-PBA@MQDs nanoparticles for different incubation times. Scale bar = 10 μm. (B) A549 cells treated with Calcein-AM and probe/NH_2_-PBA@MQDs nanoparticles, respectively. Scale bar = 20 μm.

As shown in Fig. 4A, after A549 cells were incubated with probe/NH_2_-PBA@MQDs nanoparticles for different times, the fluorescence signals of cells were significantly increased until the incubation time reached 6 h. As the incubation time gradually increased to 12 h, probe/NH_2_-PBA@MQDs nanoparticles still showed a significant fluorescence with a slightly decreased fluorescence signal intensity (Fig. S10 and Fig. S11A). This suggested that probe/NH_2_-PBA@MQDs nanoparticles were successfully uptake by A549 cells.

To further verify the specificity of this probe/NH_2_-PBA@MQDs nanoparticles for target miRNA localization, A549 cells were incubated with probe labeling PBA@MQDs nanoparticles for 6 h. The result showed that a significant fluorescence signal of PBA@MQDs nanoparticles with probe hybridization for miRNA was presented in A549 cells (Fig. 4B). These results indicated that our probe/NH_2_-PBA@MQDs nanoparticles were capable of locating and sensing miRNA in living cells with the ability of self-delivery.

**Fig. 5:**
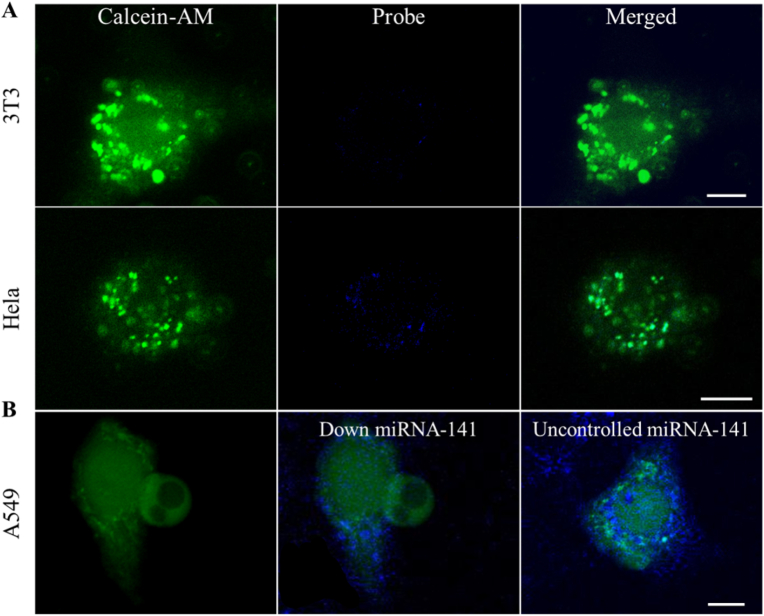
Laser scanning confocal microscopy images of (A) Calcein-AM-labeled 3T3 cells and Hela cells were co-cultured with probe/NH_2_-PBA@MQDs nanoparticles, respectively. Scale bar = 10 μm. (B) Calcein-AM-labeled A549 cells (down-regulated miRNA-141 or unregulated miRNA-141) were co-cultured with probe/NH_2_-PBA@MQDs nanoparticles. Scale bar = 10 μm.

Having performed probe/NH_2_-PBA@MQDs nanoparticles can efficiently enter A549 cells and locate intracellular miRNA, we further studied the ability of probe/NH_2_-PBA@MQDs nanoparticles to distinguish different cell lines. Therefore, we chose 3T3 cells and Hela cells to incubate with probe/NH_2_-PBA@MQDs nanoparticles in the same conditions, followed by confocal microscopy imaging. As shown in Fig. 5A, the signal for miRNA-141 in Hela cells was significant, while a negligible signal could be observed in 3T3 cells. It demonstrated that the probe/NH_2_-PBA@MQDs nanoparticles were able to distinguish different cells with levels of miRNA expression. Considering miRNA expression level is decontrolled during different stages of malignant tumors, and the sensitivity visualization of dynamic change in intracellular miRNA is of importance to given valuable diagnosis and prognosis references. Therefore, miRNA-141 inhibitors were incubated with A549 cells to down-regulated miRNA-141 expression, followed by confocal microscopy imaging. Compared with the unregulated cellular level, probe/NH_2_-PBA@MQDs nanoparticles fluorescence intensities showed a significant decrease with the lower expression level of miRNA-141 (Fig. 5B). It suggested that the probe/NH_2_-PBA@MQDs nanoparticles can achieve reliability monitor of slight fluctuations of miRNA expression level in cancer cells. These results validated that probe/NH_2_-PBA@MQDs nanoparticles were effectively delivered in cancer cells, showing efficient delivery targeting intracellular miRNA and great potential tumor diagnosis.

## 4 Conclusion

In conclusion, we demonstrated a size-controllable nanosystem-based Co-Mn PBA@MQDs with DNA probe assistance for the ultrasensitive detection and location of miRNA in living cells. Owing to the coordinate structures of Co-Mn PBAs have been controlled to regulate nucleation and growth rate during MQDs electrostatic adsorption process, allowing the formation of Co-Mn PBA@MQDs core-shell structure with homogeneous size and shape. The unique combination of bimetallic coordination organic frameworks and quantum dots endow the composite nanoparticles presented excellent electrocatalysis and fluorescence properties. The Co-Mn PBA@MQDs nanosystem loaded with DNA probe has achieved accurate selection and sensitive miRNA analysis with good stability and reproducibility. We also validated through intracellular experiments that the nanosystem processed reliable and efficient imaging of target miRNA with cell permeability and biocompatibility. This work is expected to develop a feasible strategy to precisely regulate the types of Prussian blue analogues and MXene-derived quantum dots for further biological analysis application.

## CRediT authorship contribution statement

**Qiannan You:** Writing – original draft, Visualization, Investigation, Data curation, Conceptualization. **Panyong Wang:** Investigation. **Tongtong Zhu:** Data curation. **Zixuan Jia:** Data curation. **Zhimin Chang:** Project administration. **Li Li:** Supervision, Project administration. **Wen-Fei Dong:** Writing – review & editing, Supervision, Project administration.

## Declaration of competing interest

The authors declare that they have no known competing financial interests or personal relationships that could have appeared to influence the work reported in this paper.

## Data Availability

Data will be made available on request.
