# Supplementary material for: Bifunctional MXene quantum dots-coated bimetallic Prussian blue analogues for sensitive sensing and accurate localization imaging of miRNAs in living cells
Source: Mater Today Bio. 2025 Apr 15;32:101747. doi: 10.1016/j.mtbio.2025.101747 (PMC12032912; doi:10.1016/j.mtbio.2025.101747)
Supplement: Multimedia component 1 [file mmc1.docx]

**Supplementary Material**

**Bifunctional** **MXene quantum dots-coated bimetallic Prussian blue analogues for sensitive sensing and accurate localization imaging of miRNAs in living cells**

Qiannan You ^a^, Panyong Wang ^a^, Tongtong Zhu ^a,b^, Zixuan Jia ^a,b^, Zhimin Chang ^a,b^, Li Li ^a,b*^, Wen-Fei Dong ^a,b*^

a Suzhou Institute of Biomedical Engineering and Technology, Chinese Academy of Science, Suzhou 215163, P. R. China

b School of Biomedical Engineering (Suzhou), Division of Life Sciences and Medicine, University of Science and Technology of China, Hefei 230026, P. R. China

Corresponding Author

^*^ E-mail: lil@sibet.ac.cn; [wenfeidong@sibet.ac.cn](mailto:wenfeidong@sibet.ac.cn).

S1. Materials

Ti_2_AlC powder (99%, 200 mesh) was purchased from 11 Technology Co., Ltd. (Jilin, China). Manganese sulfate (MnSO_4_) and Potassium hexacyanocobaltate (III) (K_3_[Co(CN)_6_]) were obtained from Aladdin Bio-Chem Technology Co., Ltd. (Shanghai, China). Lithium fluoride (LiF) was obtained from Aladdin Biochemical Technology Co., Ltd. (Shanghai, China). Hydrochloric acid (HCl) and potassium chloride (KCl) were obtained from Lingfeng Chemical Reagent Co., Ltd. (Shanghai, China). Ammonium hydroxide (NH_3_·H_2_O) and Polyetherimide (PEI) were purchased from Greagent Co., Ltd. (Shanghai, China). 1-ethyl-3-[3-(dimethylamino) propyl] carbodiimide hydrochloride (EDC) and N-hydroxysuccinimide (NHS) were obtained from Adamas Reagent Co., Ltd. (Shanghai, China). Calcein-AM was obtained from ThermeoFisher Scientific Co., Ltd. (Shanghai, China). 3-(4,5-dimethylthiazol)-2,5-diphe-nyltetrazolium bromide (MTT), Roswell Park Memorial Institute 1640 (RPMI-1640), penicillin-streptomycin solution, Fetal bovine serum (FBS) and 4% paraformaldehyde were purchased from Beyotime Institute of Biotechnology (Shanghai, China). All the chemicals are of analytical reagent grade or better and all aqueous solutions were prepared with deionized (DI) water. All the sequences of DNA oligonucleotides used in this work were obtained from Sangong Biotech Co., Ltd. (Shanghai, China) and listed in Table S1.

S2. Apparatus

Transmission electron microscopy (TEM) images were gained on an FEI Talos F200s instrument. Scanning electron microscope (SEM) images were obtained on a Gemini-SEM 300 instrument. X-ray diffraction (XRD) patterns were characterized by a D/MAX 2500 V/PC with a Cu-Ka line (0.15419 nm). Fourier transform infrared spectra (FT-IR) were obtained on a Thermo Scientific Nicolet iS20. X-ray photoelectron spectroscopy (XPS, ESCLAB MKII) was carried out on a Thermo Scientific K-Alpha Raman spectra obtained on a LabRam HR Evolution instrument. Zeta potential conducted potential tests on the Zetasizer Nano ZS90. Ultraviolet absorption spectroscopy (UV-vis) was performed on a U-3900H spectrophotometer, and Thermogravimetric curves (TG) were analyzed by a Netzsch STA 449F3 instrument. The specific surface area of the samples was calculated from the nitrogen adsorption isotherms measured with a BK200B-01-Analysis based on the Brunauer-Emmett-Teller (BET) model. Fluorescence spectra were obtained by an F97 Pro fluorescence spectrophotometer. Confocal laser scanning microscope (CLSM) images were obtained on a Leica TCS SP5 II instrument.

S3. Electrochemical measurements

All electrochemical experiments were performed with a CHI660E electrochemical workstation (Shanghai Chenhua Instrument Co., Ltd., China) with a three-electrode system, including a miRNA/probe/NH_2_-PBA@MQDs working electrode, a platinum wire as the counter electrode, and a saturated silver-silver chloride electrode (Ag/AgCl) reference electrode. For the cyclic voltammetry (CV) test, the working type was set to the normal mode with the scan rate of 50 mV/s from -0.1 V to 0.6 V in 10 mM K_3_[Fe(CN)_6_] solution containing 0.1 M KCl. Square wave voltammetry (SWV) was carried out with a potential step of 5 mV, a frequency of 5 Hz, and an amplitude of 25 mV. Electrochemical impedance spectroscopy (EIS) was obtained in a 5 mM [Fe(CN)_6_]^3-/4-^ solution containing 0.1 M KCl with a frequency ranging from 0.1 Hz to 10^5^ Hz, and an amplitude of 5 mV.


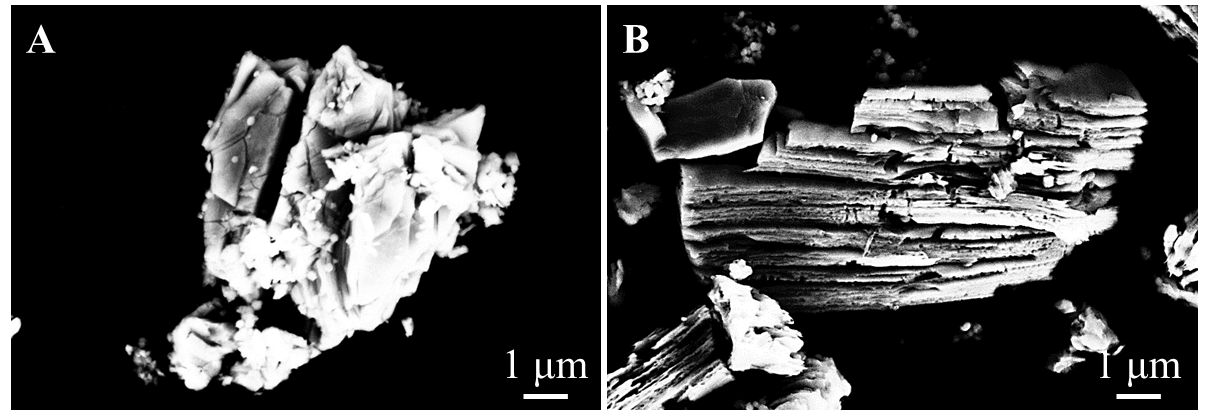


Fig. S1. SEM images of (A) Ti_2_AlC MAX, and (B) Ti_2_CT_x_ MXene etching for 36 h.


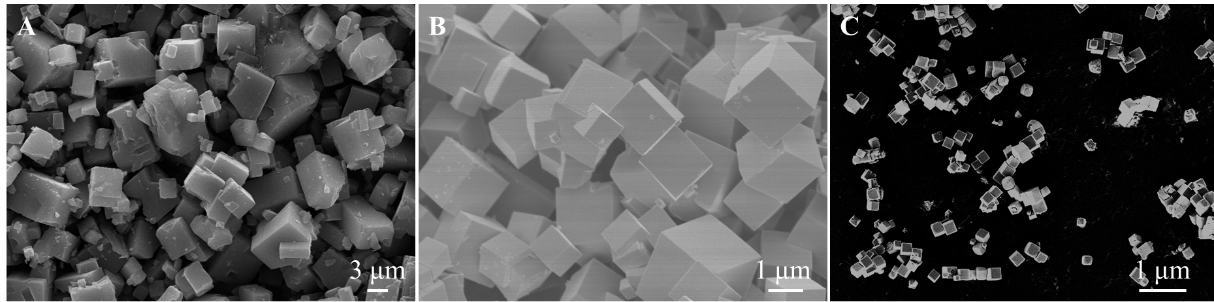


Fig. S2. SEM images of Co-Mn PBA nanoparticles with different injection rates (A) 1000 μL/min, (B) 500 μL/min, and (C) 200 μL/min.


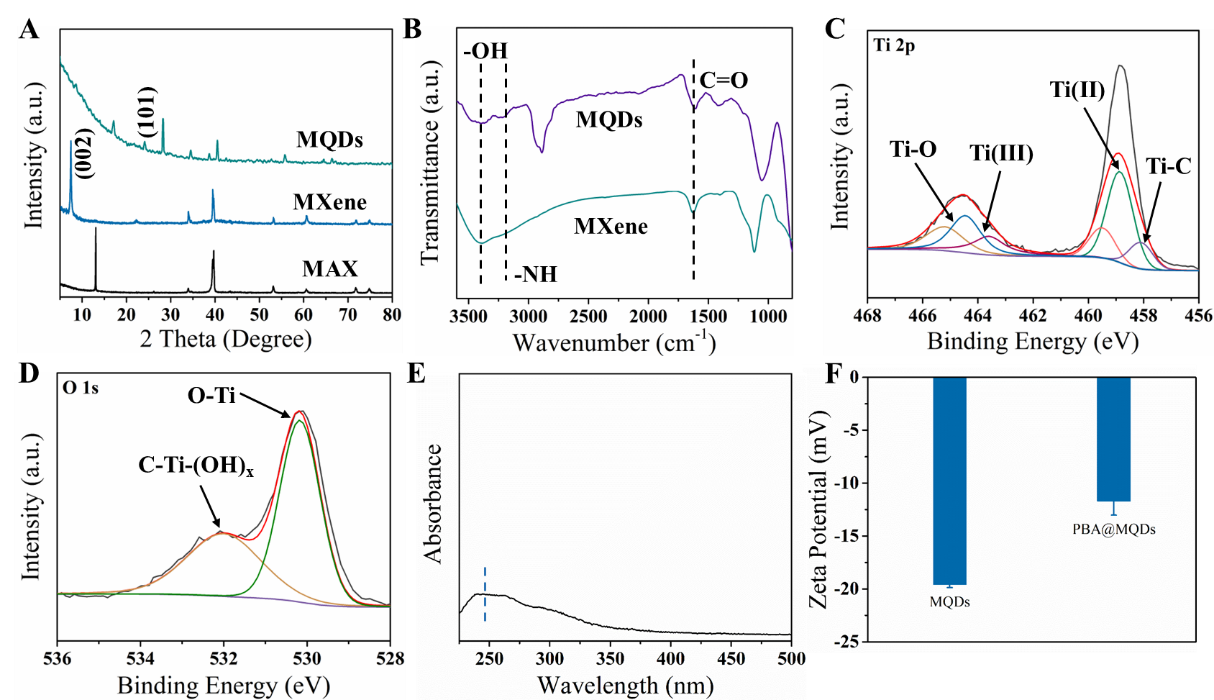


Fig. S3. (A) XRD patterns of Ti_2_AlC MAX, Ti_2_CT_x_ MXene, and MQDs; (B) FT-IR spectra of Ti_2_CT_x_ MXene and MQDs; XPS spectra of MQDs: (C) Ti 2p, and (D) O 1s; (E) UV-vis absorption spectrum of the MQDs. (F) ζ-Potential of MQDs and PBA@MQDs.


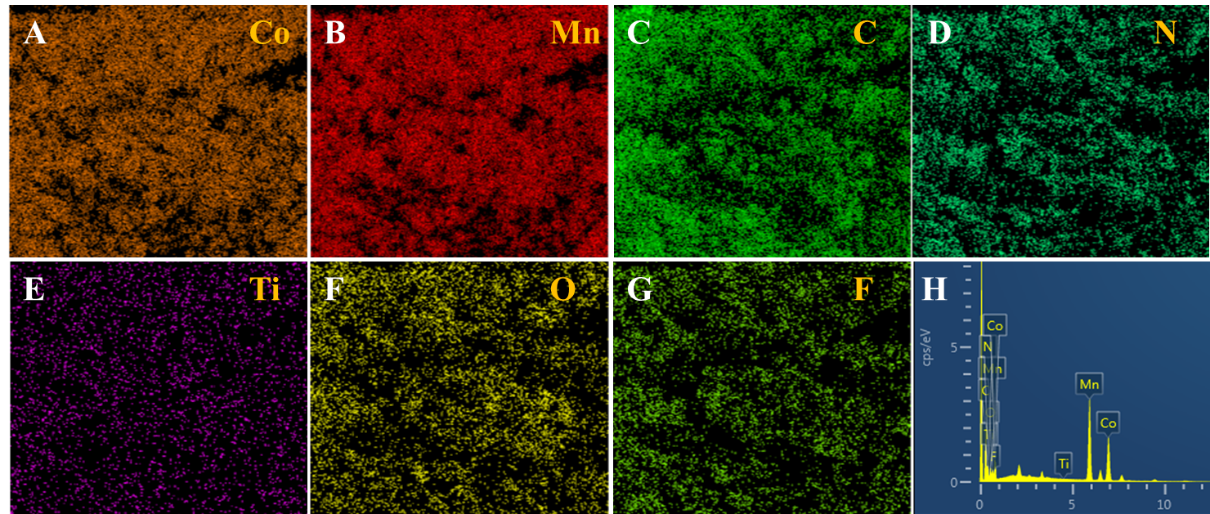


Fig. S4. The corresponding elemental mapping images (A-G) and EDS spectrum (H) of PBA@MQDs.


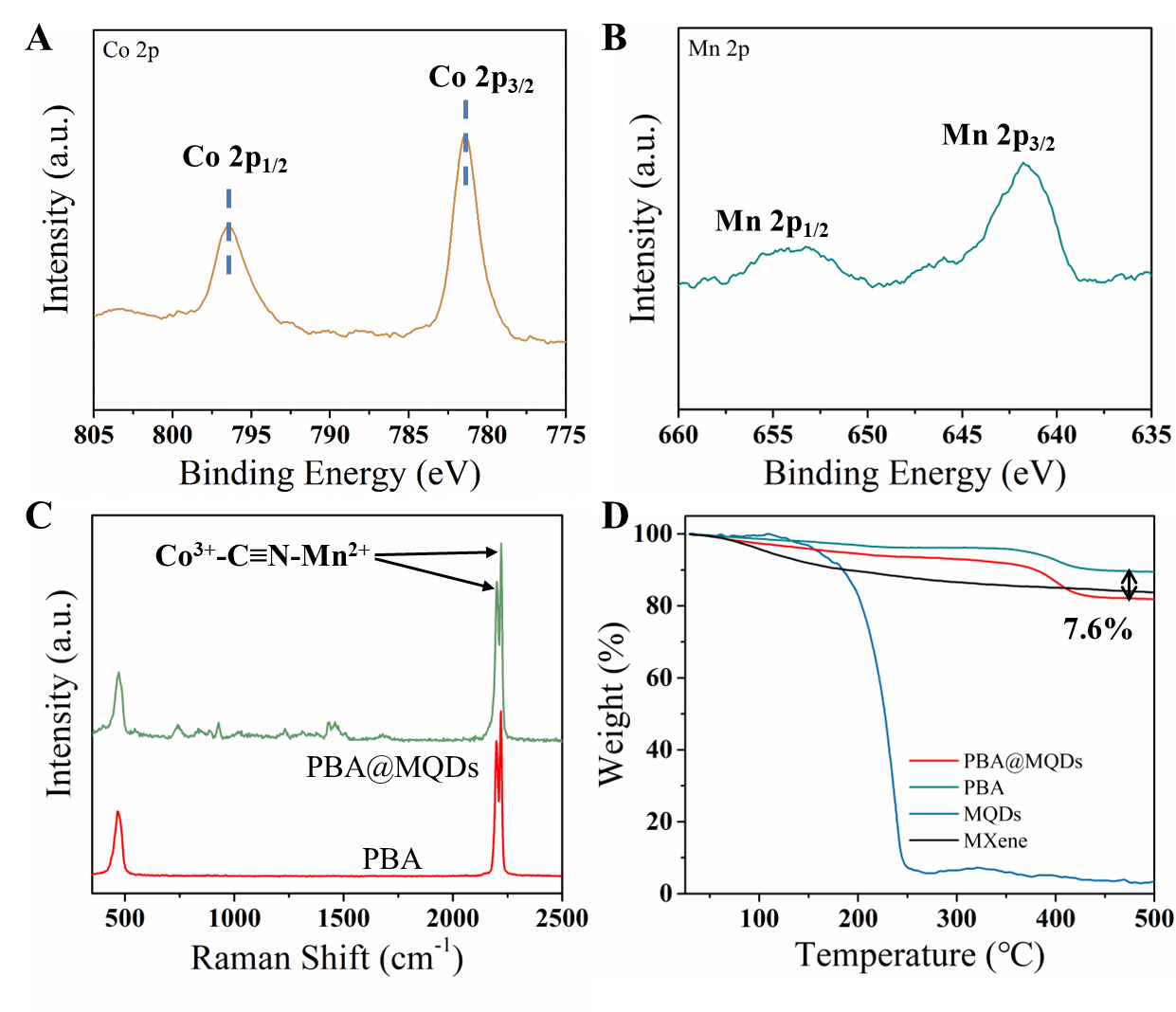


Fig. S5. XPS spectra of PBA@MQDs of (A) Co 2p and (B) Mn 2p; (C) Raman spectra of PBA and PBA@MQDs. (D) TG analyses of the Ti_2_CT_x_ MXene, MQDs, PBA, and PBA@MQDs. The samples were tested under an N_2_ atmosphere at a heating rate of 5 ℃/min.


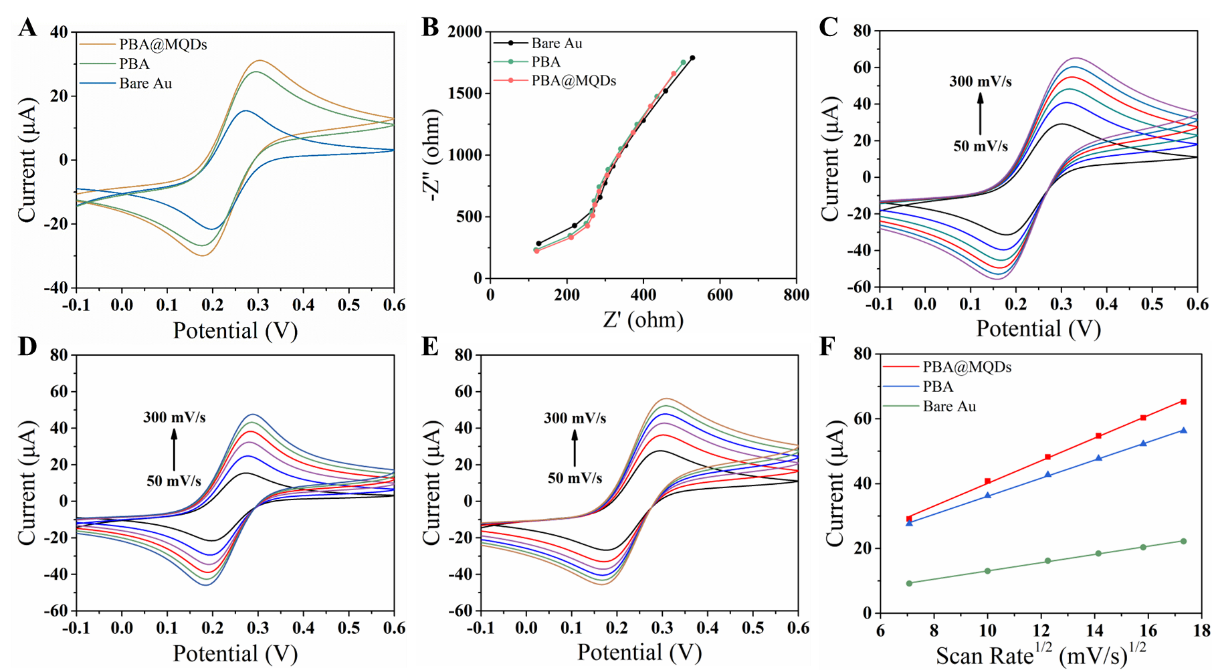


Fig. S6. (A) CV behaviors and (B) EIS measurements of bare Au disk electrode, PBA, and PBA@MQDs modified electrodes. The solution of CV contains 10 mM K_3_[Fe(CN)_6_] and 0.1 M KCl; (C-E) CV curves of the bare Au disk electrode, PBA, and PBA@MQDs modified electrodes at different scan rates from 50 mV/s to 300 mV/s, respectively. The system of CV contains 10 mM K_3_[Fe(CN)_6_] and 3 M KCl; (F) Calibration curves for the peak current vs. the square root of the scan rate for bare Au disk electrode, PBA, and PBA@MQDs modified electrodes, respectively.


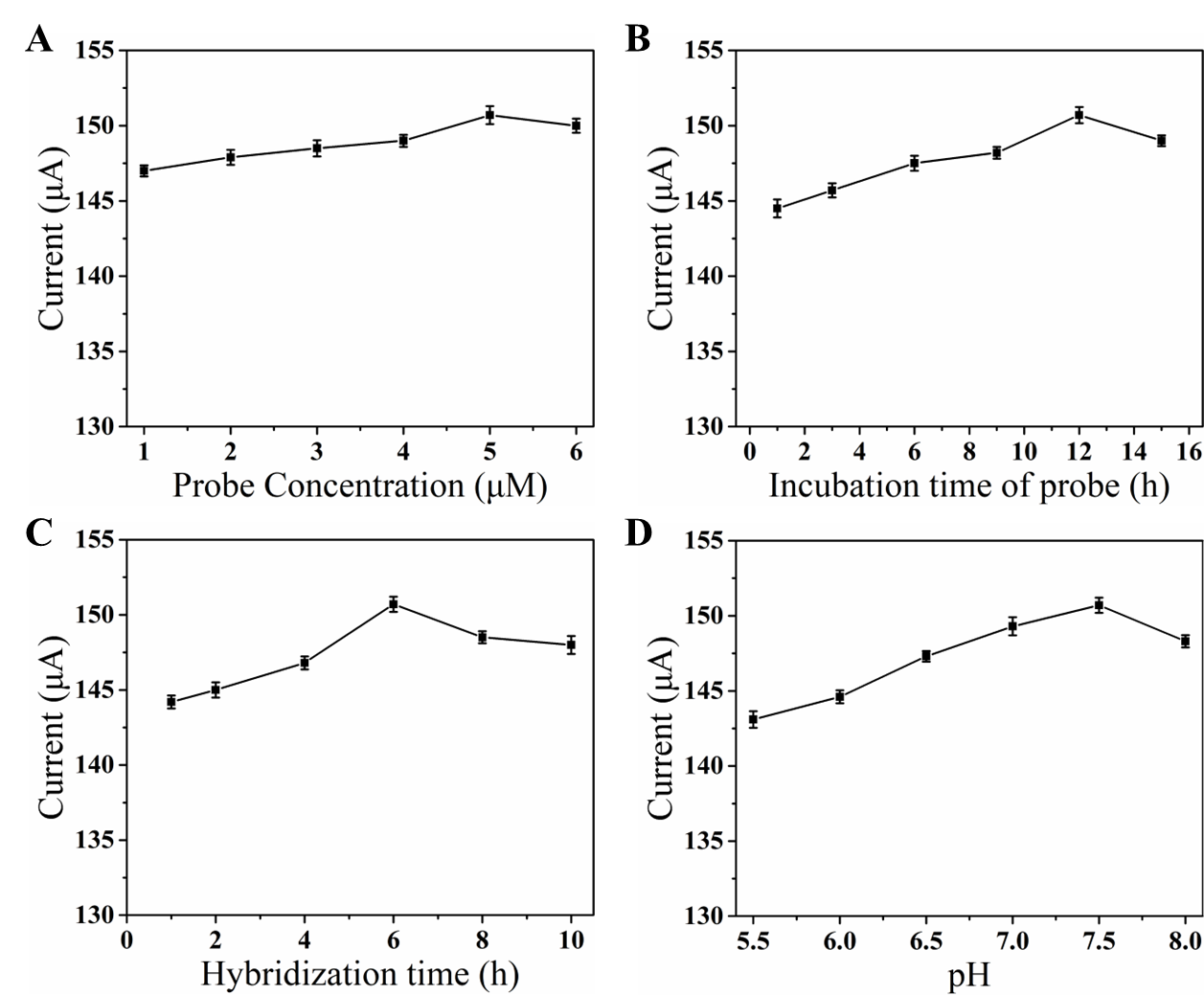


Fig. S7. The effect of different parameters on the biosensing performance of the as-prepared biosensor by SWV responses. (A) The concentration of probe; (B) The incubation time of NH_2_-PBA@MQDs modified electrode with probe; (C) The hybridization time of miRNA-141 with probe/NH_2_-PBA@MQDs modified electrode; (D) The pH value of H-buffer. The concentration of miRNA-141 is 1 fM.


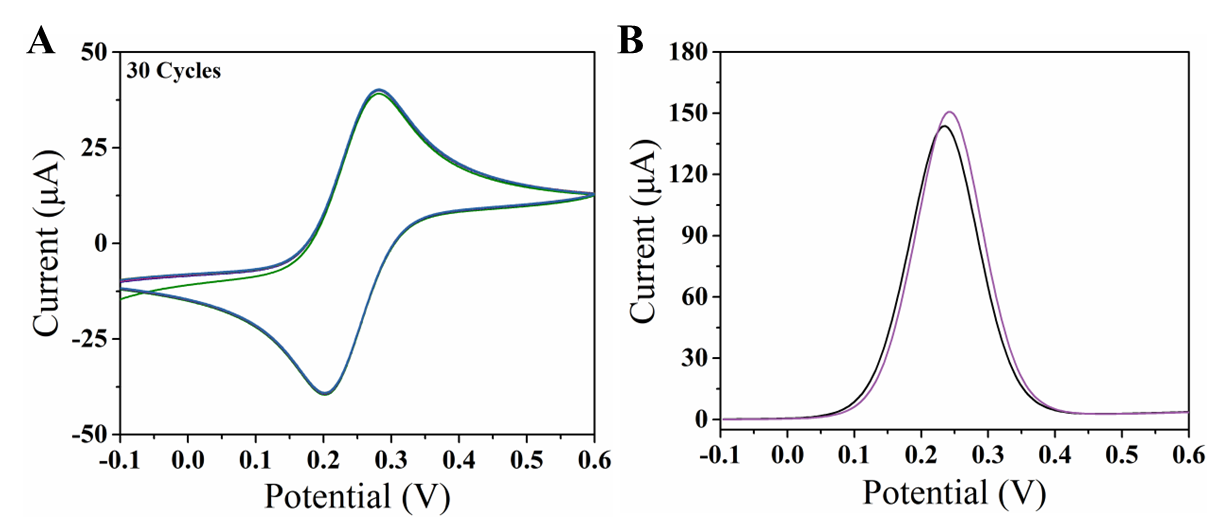


Fig. S8. (A) Stability of the biosensor scanning 30 times in the presence of 10 mM K_3_[Fe(CN)_6_] containing 0.1 M KCl; (B) SWV response of the biosensor with and without miRNA-141. The concentration of miRNA-141 is 1 fM.





Fig. S9. Cytotoxicity assays of the PBA@MQDs against A549, Hela, and 3T3 cells, respectively.


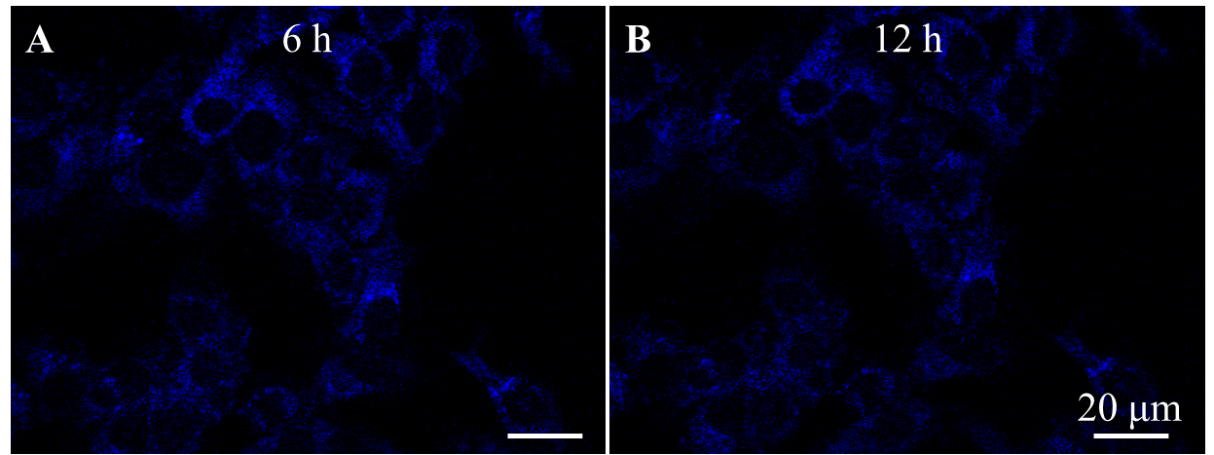


Fig. S10. A549 cells treated with probe/NH_2_-PBA@MQDs nanoparticles for different incubation times (A) 6 h, (B) 12 h.


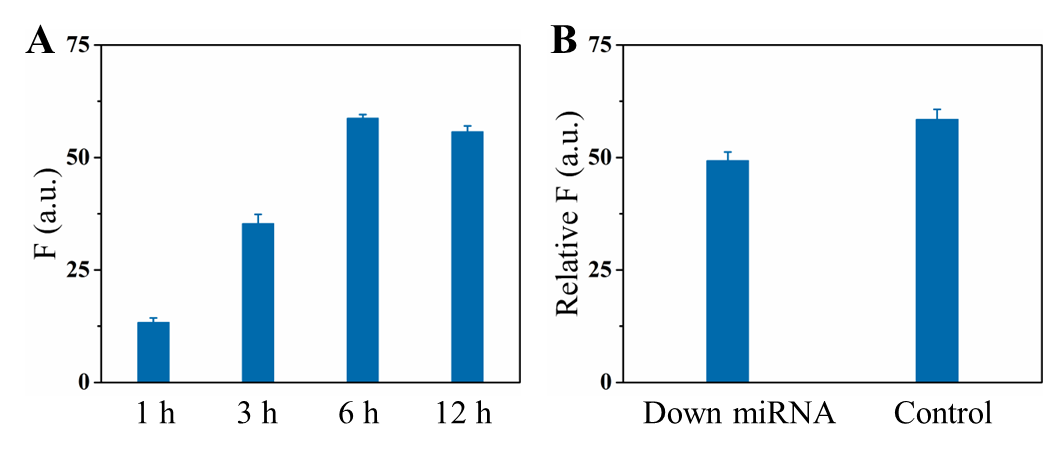


Fig. S11. (A) The relative fluorescence intensity of A549 cells treated with probe/NH_2_-PBA@MQDs nanoparticles for different incubation times. (B)The corresponding relative fluorescence intensities of Calcein-AM-labeled A549 cells (down-regulated miRNA-141 or unregulated miRNA-141) were co-cultured with probe/NH_2_-PBA@MQDs nanoparticles.

Table S1. Sequences of DNA oligonucleotides are used in this work.

| miRNA-141 | 5′-UAA CAC UGU CUG GUA AAG AUG G-3′ |
| --- | --- |
| probe | 5′-COOH-CCA TCT TTA CCA GAC AGT GTT A-3′ |
| miRNA-200a | 5′-UAA CAC UGU CUG GUA ACG AUG U-3′ |
| miRNA-155 | 5′-UUA AUG CUA AUC GUG AUA GGG GU-3′ |
| miRNA-21 | 5′-UAG CUU AUC AGA CUG AUG UUG A-3′ |
| miRNA-182 | 5′-UUU GGC AAU GGU AGA ACU CAC ACU-3′ |
| miRNA-200b | 5′-UAA UAC UGC CUG GUA AUG AUG A-3′ |

Table S2. Various buffers are used in this work.

| W-buffer | 10 mM Tris-HCl | pH=7.4 |
| --- | --- | --- |
| I-buffer | 10 mM Tris-HCl, 1 mM EDTA, 0.1 M NaCl | pH=7.4 |
| H-buffer | 10 mM Tris-HCl, 1 mM EDTA, 0.2 M NaCl | pH=7.4 |
| MES buffer | 0.1 M MES, 0.5 M NaCl | pH=6.0 |

Table S3. Comparison of the detection performance for the miRNA with other reported work.

| **Materials** | **Techniques** | **Linear range** | **LOD** | **Reference** |
| --- | --- | --- | --- | --- |
| Ru-MOF-sDNA/Fe_3_O_4_@SiO_2_@Au-cDNA | ECL | 1 fM ~ 10 pM | 0.3 fM | [1] |
| 3D walker | ECL | 10 fM ~ 100 pM | 3.3 fM | [2] |
| AuNPs-DNA/cDNA-MEA/Pdots | PEC | 1 fM ~ 10 pM | 0.5 fM | [3] |
| HP4-CdS QDs/Exo III/output DNA | PEC | 1 fM ~ 1 nM | 0.33 fM | [4] |
| 2D walker | fluorescence | 100 fM ~ 1 nM | 58 fM | [5] |
| AgNCs/HpDNA | SPR | 0 ~ 50 pM | 1 fM | [6] |
| AuNU/PVP/CS-GO | EC | 2 fM ~ 0.5 μM | 0.94 fM | [7] |
| PBA@MQDs | EC | 1 fM ~ 1 nM | 0.37 fM | This work |

Table S4. Measure of miRNA-141 added in serum samples with the proposed biosensor.

| **Serum**  **sample** | **Add concentration** | **Founded concentration** | **Recovery (%)** | **RSD**  **(%)** |
| --- | --- | --- | --- | --- |
| 1 | 10 pM | 9.56 pM | 95.6 | 2.3 |
| 2 | 0.1 pM | 0.097 pM | 97.0 | 1.9 |
| 3 | 1 fM | 1.012 fM | 101.2 | 2.6 |

**References**

[1] H. Shao, J. Lu, Q. Zhang, Y. Hu, S. Wang, Z. Guo, Sensors and Actuators B: Chemical, 268 (2018) 39-46.

[2] Z. Xu, L. Liao, Y. Chai, H. Wang, R. Yuan, Analytical Chemistry, 89 (2017) 8282-8287.

[3] N. Zhang, X.-M. Shi, H.-Q. Guo, X.-Z. Zhao, W.-W. Zhao, J.-J. Xu, H.-Y. Chen, Analytical Chemistry, 90 (2018) 11892-11898.

[4] X. Niu, C. Lu, D. Su, F. Wang, W. Tan, F. Qu, Analytical Chemistry, 93 (2021) 13727-13733.

[5] L. Wang, R. Deng, J. Li, Chemical Science, 6 (2015) 6777-6782.

[6] J. Zhang, C. Li, X. Zhi, G.A. Ramón, Y. Liu, C. Zhang, F. Pan, D. Cui, Analytical Chemistry, 88 (2016) 1294-1302.

[7] A. Khodadoust, N. Nasirizadeh, R.A. Taheri, M. Dehghani, M. Ghanei, H. Bagheri, Microchimica Acta, 189 (2022) 213.
